# Retraction: Anti-Tumor Activity of a Novel Compound-CDF Is Mediated by Regulating miR-21, miR-200, and PTEN in Pancreatic Cancer

**DOI:** 10.1371/journal.pone.0205300

**Published:** 2018-10-02

**Authors:** The PLOS ONE Editors

After publication of this article [1], similarities were noted between the following pairs of images in Fig 5C:

- Second AsPC-1 panel (GEM 20 nmol/L) and first CDF-pre-treated AsPC-1 panel (GEM 0 nmol/L)
- Fourth and sixth AsPC-1 panels (GEM 40 nmol/L and CDF 6 μmol/L)
- Fourth and seventh CDF-pre-treated AsPC-1 panels (GEM 40 nmol/L and CDF 8 μmol/L)

Wayne State University investigated this work and confirmed that the three images were duplicated in the indicated figure panels. The institution further noted concerns about record-keeping and data management pertaining to this experiment, including questions as to whether the laboratory records align with experimental details and data reported in the article.

In light of these issues, the *PLOS ONE* Editors retract this article due to concerns about the reliability of the reported data and the accuracy with which results were reported.

BB agreed with the retraction. SA, DK, SHS, ZW, SB, AA, SP, PAP and FHS did not respond.
